# Shifting the Paradigm of Emergency Care in Developing Countries

**DOI:** 10.7759/cureus.2219

**Published:** 2018-02-22

**Authors:** Ayesha Khan, Bradley T Penoff, Elizabeth A Pirrotta, Robert Hosang

**Affiliations:** 1 Emergency Medicine, Stanford University School of Medicine; 2 Payments, Google Inc; 3 Department of Emergency Medicine, Stanford University School of Medicine; 4 School of Public Health, University of California, Berkeley

**Keywords:** international medicine, epidemiology, burden of disease, global health

## Abstract

Background

The global agenda does not address a significant amount of preventable death in low- and middle-income countries (LMICs). While illnesses requiring acute care are increasing at an alarming rate in these countries, there are inadequate numbers of physicians or nurses to deal with the growing burden. Many people feel that emergency systems are too expensive and restricted in scope to have public health implications in resource-limited areas. Little empirical data exists to suggest otherwise. The goal of this study was to delineate the type and frequency of emergency conditions and define a novel method to estimate the burden of emergency diseases in Fort Liberte, Haiti.

Methods

A retrospective, cross-sectional medical record review was performed on all emergency room visits to Fort Liberte Hospital in 2009 and 2010. The type, frequency, and annual incidence of emergency conditions were identified and used to determine the burden of emergency disease. A disability-adjusted life year (DALY) calculation was estimated using a variation on a model of indirect national data extrapolation to cities.

Results

Nineteen months of data available yielded 2000 charts with 2284 diagnoses in total. Trauma was the most common illness at 13% of all charts, followed by abdominal pain at 11%, gastroenteritis at 8%, skin and soft tissue infections at 7%, and hypertension at 6%. The DALY calculation showed disability from emergency conditions to be five times that of HIV, malaria, and TB combined.

Conclusions

Sufficient emergency burden of disease affects population health in Fort Liberte, Haiti to warrant addressing it as a public health concern. The kinds of conditions described in this review may be amenable to task shifting as a feasible, sustainable, and scalable way to address the burden in a cost-effective manner.

## Introduction

Global health agencies historically forego development of emergency systems in favor of primary care as a low-cost way to provide care for the most people in low- and middle-income countries (LMICs) [1]. However, as countries develop, disease burden is shifting away from communicable disease toward accidents, injuries, and non-communicable disease, and emergency programs can no longer be considered non-essential in international health development [2].

The World Health Organization (WHO) allocates resources for the development of health programs by the disease-based assessment of mortality and disability from 107 diseases and injuries [3-7]. This assessment, termed as the Global Burden of Disease (GBD), measures the burden of disease in a population by calculating the disability-adjusted life year (DALY), the number of years of life lost in a population due to ill health, disability, or death. Each DALY calculation requires a disability weighting that reflects the severity of the number of years the person is affected. Since emergency illnesses are limited and may evolve into death, chronic conditions, or subacute condition, there is not a “years” to put into the calculation. Emergency medicine is systems based, providing care across a spectrum of diseases [1] and the emergency portion of diseases like diabetes or hypertension is obscured in the overall DALYs for the chronic conditions. The inability to separate and quantify emergency processes for a vast number of diseases obscures the need for emergency care in a population. This lack of quantitative data has been proposed to be one reason that developing emergency health systems have been overlooked in LMICs. Further, many feel that despite the apparent increasing burden of emergency disease, emergency systems are too expensive, individualistic, and restricted in scope to have public health implications in resource-limited areas. In this paper, we examine if emergency care should be addressed as a public health concern in Fort Liberte, Haiti by 1) examining the type and frequency of illnesses seen in the emergency room, 2) using a novel methodology to derive an absolute DALY of emergency conditions, and 3) comparing this DALY of emergency conditions to that from human immunodeficiency virus (HIV), tuberculosis (TB), and malaria, the most internationally well-funded diseases in the mid-size Haitian town of Fort Liberte [8,9].

## Materials and methods

Study design

A retrospective cross-sectional medical record review was performed to estimate the burden of emergency disease and to assess the type and frequency of visits to the acute care area of Fort Liberte Hospital (FLH). A DALY calculation was indirectly estimated by extrapolating population data from the Haitian town of Fort Liberte. The Committee for the Protection of Human Subjects at the University of California, Berkeley approved this study with a waiver of informed consent.

Study setting

Fort Liberte is a mid-size Haitian town with a population of 28,000. Its economy is driven by fishing, small business, and farming. Three medical facilities serve this town: FLH and an attached clinic that are for-profit, and a church-based clinic [10]. The hospital sees approximately 150 patients per day, 5-10% of whom are classified as “emergency” cases based on self-triage or re-direction from health staff based on visible distress of the patient. There is one operating theater, no blood bank, no monitored beds, and no oxygen available. Healthcare providers in the hospital are not trained in emergency care and a doctor is not always present in the emergency room (ER). One doctor staffs the entire hospital from 3 pm to 7 am. Pediatrics, internal medicine, surgical, and obstetric services are available in the hospital. The church-based clinic is staffed for two weeks quarterly by the United States primary care providers.

Anecdotally, there are a moderate number of acute injuries from fishing in ill-equipped boats, road traffic accidents, and meager attempts at farming with poor equipment. Often, lacerations and fractured limbs are left to heal with no medical attention.

Study protocol

All charts coded as ER visits from the years 2009 to 2010, approximately one year before the Haiti earthquake and one year after, were queried from FLH. All-comers seen in the ER have charts coded as ER visits. Available months of records are shown in Table 1.

**Table 1:** Emergency charts, Fort Liberte hospital (FLH). X: Available records O: Records missing or destroyed

Emergency charts FLH		
	2009	2010
January	O	X
February	X	O
March	X	X
April	X	O
May	X	O
June	X	O
July	X	X
August	X	X
September	X	X
October	X	X
November	X	X
December	X	X

An American graduate student and a Creole-English speaking translator initially reviewed the charts for inclusion based on the following criteria: 1) patient presentation to the ER and 2) occurrence within the years 2009 and 2010. Subsequent review by the principal investigator with a Creole-English translator was performed. Age, sex, diagnosis, and when available, disposition were recorded in Excel (Microsoft, Redmond. WA).

Due to variability in the doctors staffing the ER and inconsistent naming of diagnoses, an *a priori* grouping was decided by the primary investigator as shown in Table 2.

**Table 2:** Composite diagnosis.

Composite diagnosis	Diagnosis from chart
Skin and soft tissue (SST)	Laceration, wound, wound infection, burns, abrasion, abscess, zoster, lesions
Difficulty in breathing (DIB)	Dyspnea, respiratory distress, asthma
Trauma	Trauma, motor vehicle collision, extremity trauma, domestic abuse, polytrauma, trauma NOS, abdominal trauma
Anemia	Severe anemia, decompensated anemia, moderate anemia
Respiratory infection	Respiratory infection, cough, bronchitis, pneumonia
Abdominal pain	Abdominal pain, epigastric pain, gastritis, gastric ulcer attack, umbilical pain
Gastroenteritis	Diarrhea, vomiting, parasites, gastroenteritis
Cardiac	Cardiopathy, chest pain, cardiac insufficiency
Gynecological complaint (GYN)	Vaginal discharge, pelvic inflammatory disease (PID), trichomonas vaginitis, vaginal infection, yeast vaginitis, vaginal bleed, dysmenorrhea

Data analysis

The frequency of diagnoses and the demographics of patients were analyzed using SAS version 9.2. After the top diagnoses were tabulated, DALYs were estimated. Calculating DALYs directly requires quantifying the incidence of disease, mortality rates, weighting of the disability caused by the disease, and the disease course. Thus, direct calculation is beyond the scope of this study. Instead, we chose to calculate DALYs indirectly. Indirect calculation allows us to estimate the proportion of emergency DALYs for a particular disease set [10]. We used the burden of disease found in Haiti in the 2004 GBD and divided these DALYs by the population of Haiti to get the DALY per capita for each disease [9]. We multiplied the yearly incidence of emergency cases in Fort Liberte by the DALY per capita of that diagnosis in Haiti to determine the DALY of emergency presentations of each disease in Haiti. We accounted for five months of missing data by dividing the total of each diagnosis by 19 and multiplying by 12 to estimate a yearly incidence of emergency cases in Fort Liberte for each diagnosis. See Appendix 1 for details.

## Results

**Figure 1:**
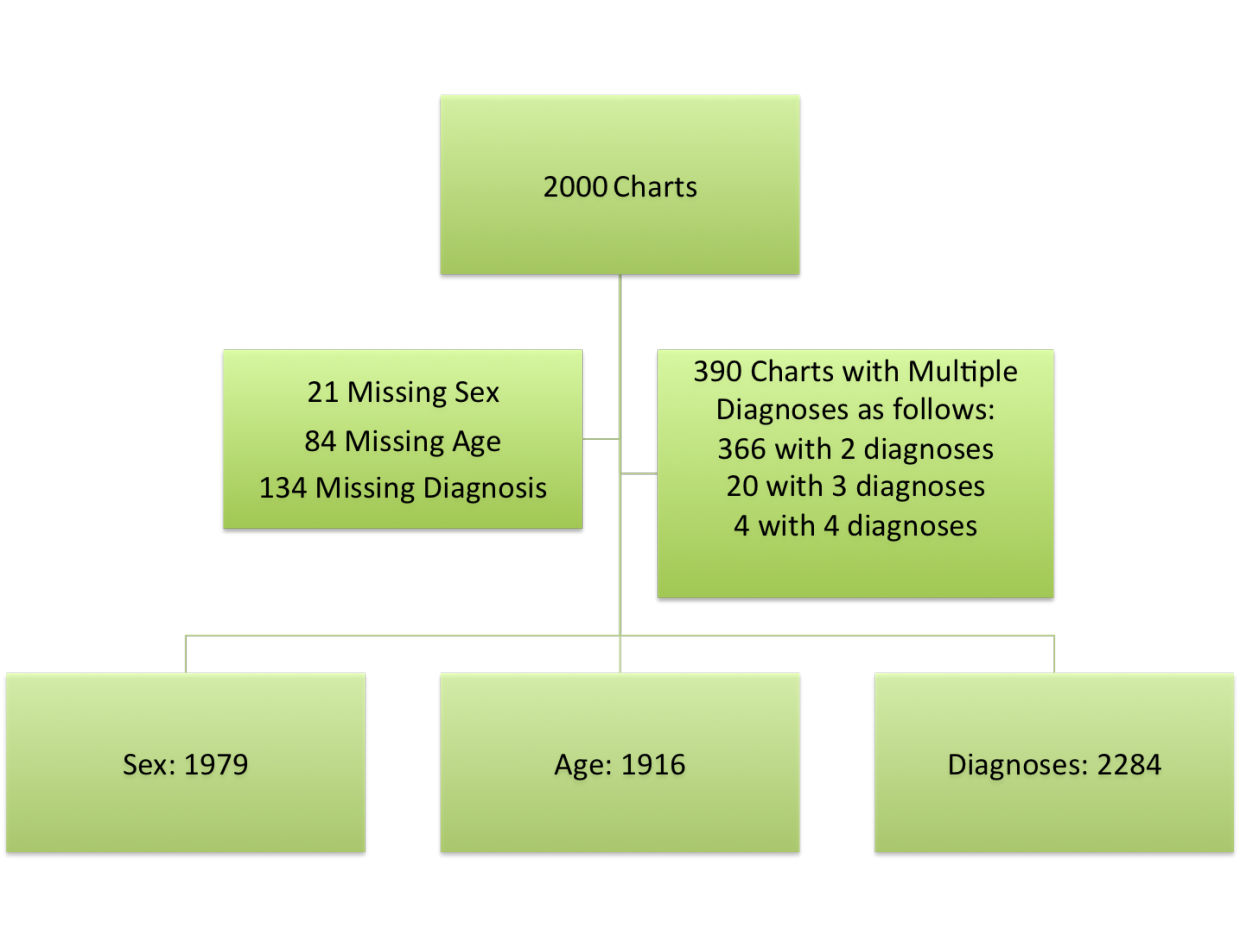
Available data from 2009 to 2010 emergency room charts at Fort Liberte hospital.

One hundred and thirty-four of the charts lacked diagnostic information or the information was illegible and three hundred and ninety charts had multiple diagnoses as described in Figure 1, resulting in 2000 charts included for analysis.

In total, 1,148 of the diagnosis fit into one of these nine composite categories; the remaining 1,136 were given their own category.

**Figure 2:**
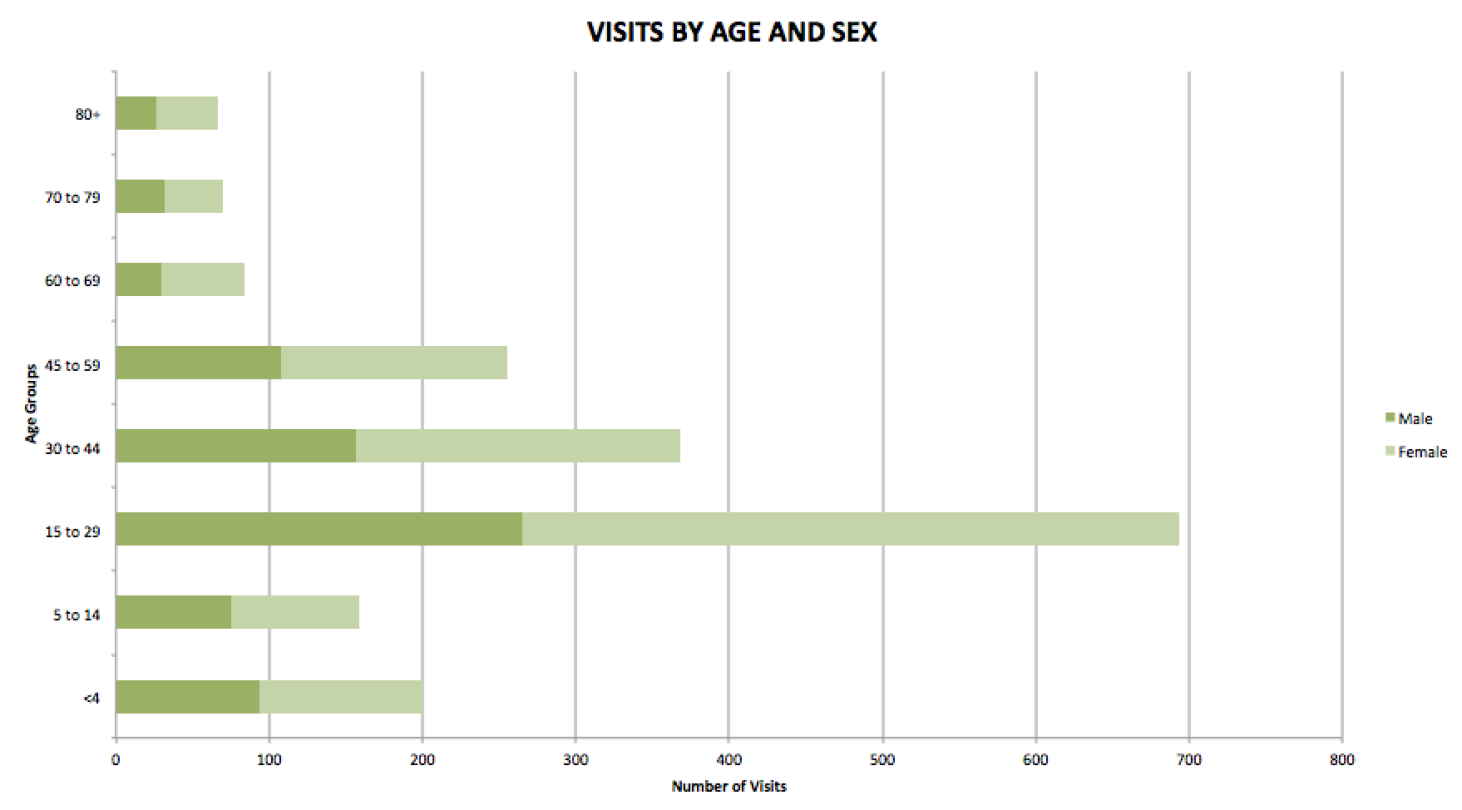
Emergency room visits to Fort Liberte hospital (2009-2010).

Figure 2 summarizes visits to the ER by age and sex followed by a more detailed description of visitors to the ER and households surveyed.

More women visited the ER than men at a ratio of 3:2. The age group that most visited the ER was that between the ages of 15 and 29 (37% of patients). The next most frequent age groups were 30-44 and 45-59.

The most frequent diagnoses of the ER visits are listed in Table 3.

**Table 3:** Most common emergency diagnoses at Fort Liberte hospital.

Top diagnoses at FORT LIBERTE hospital		
Diagnosis	Visits	Percent
Trauma	290	13%
Abdominal pain	255	11%
Gastroenteritis	187	8%
Skin and soft tissue	164	7%
Hypertension	104	6%
Anemia	87	4%
Typhoid	87	4%
Respiratory infections	87	4%

Trauma was the most common reason to visit the ER, with 60% of trauma cases caused by road traffic accidents. Eight percent of the trauma cases were further diagnosed as long bone fractures. Fourteen percent of the trauma cases involved closed head injuries. Although women comprised most of the overall visits, men dominated the trauma patients with 56% of visits. Forty-four percent of the trauma visits were in the 15- to 29-year-old age group.

Lacerations were not included in the trauma category but rather in skin and soft tissue (SST), because the treatment protocol for SST complaints is similar (wound care rather than trauma life support). Twenty-nine percent of the SST complaints were lacerations, with a male predominance of 4:1; 41% of SST complaints were wounds.

Upper abdominal pain had a largely female predominance of 3:1. Anemia also had a female predominance of 2:1. Forty-eight percent of all anemias were in the 15-44-year-old group. Gastroenteritis (comprised of 70% diarrhea and 29% intestinal parasite cases) was a disease of the young, with a quarter of cases being under the age of four and another 34% being between 15 and 29 years old.

Upper and lower respiratory infections comprised 4% of all visits. Of these, 47% were in children four years old and under. Toxic ingestions also occurred predominately in children, with 67% under the age of four.

**Figure 3:**
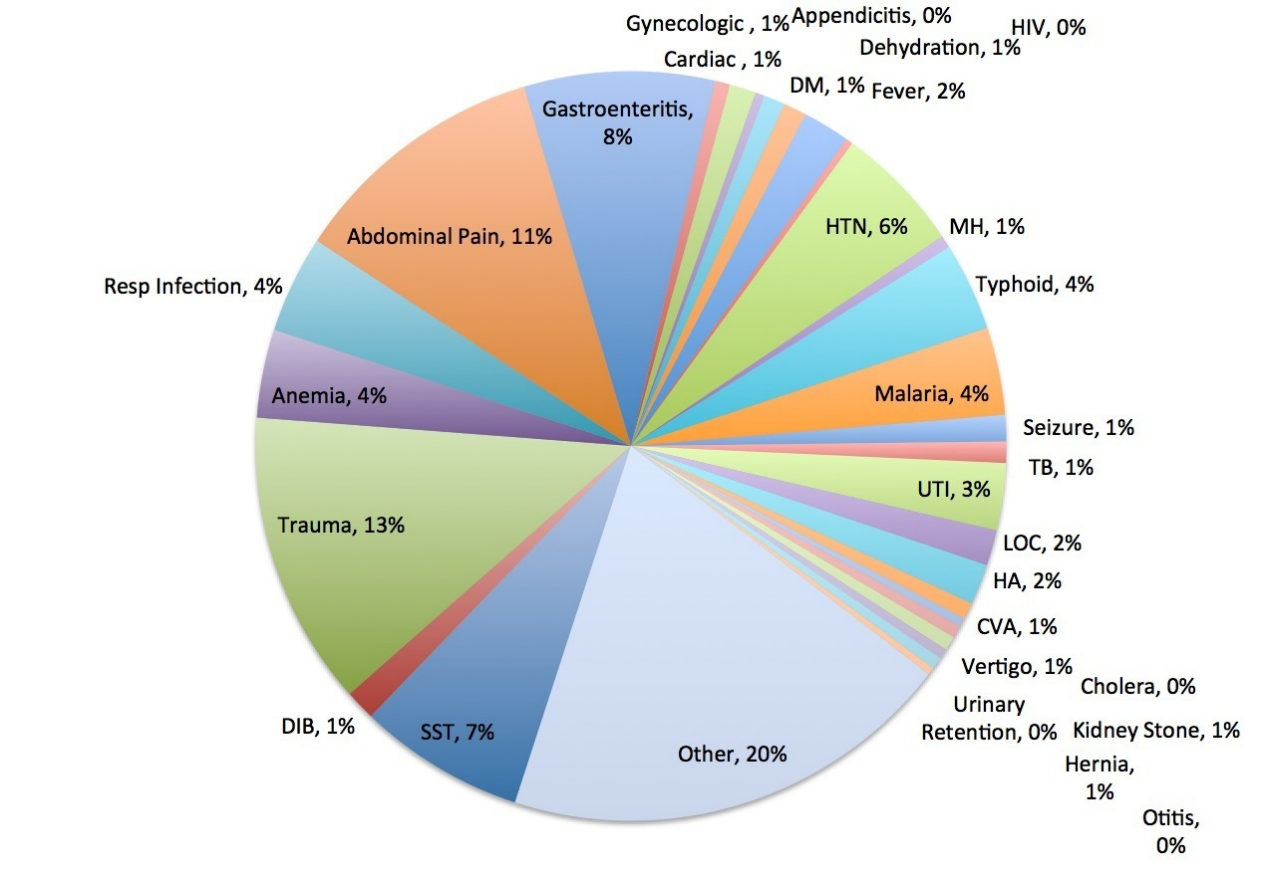
Emergency room diagnoses (2009-2010) at Fort Liberte hospital. CVA: Cerebrovascular accident; DIB: Difficulty in breathing; HA: Headache; HIV: Human immunodeficiency virus; LOC: Loss of consciousness; MH: Mental health; SST: Skin and soft tissue infection; TB: Tuberculosis; UTI: Urinary tract infection.

Fever as a complaint was evenly distributed across sexes and fairly evenly distributed across age groups, with 24% in the less than a four-year-old group, 24% in the 15-29 years old group, and 20% in the 30-44 years old group. Dehydration most affected vulnerable populations with 30% under four years old and 40% over 60 years old. Figure 3 shows ER diagnoses for 2009 and 2010 at FLH.

Table 4 shows the estimated annual DALYs of emergency presentations in Fort Liberte.

**Table 4:** Estimated emergency disability adjusted life years/year at Fort Liberte hospital.

Diagnosis	Estimated emergency DALYs per year in Fort Liberte
TB	0.15
Malaria	0.08
Respiratory infections	2.55
Genitourinary	0.00
Diarrheal disease	1.99
Intestinal nematode infection	0.04
HIV	0.11
Birth asphyxia	0.00
Other infectious	2.51
Anemia	0.73
Diabetes mellitus	0.06
Oral conditions	0.01
Respiratory diseases	0.13
Hypertensive heart disease	2.84
Neuropsychiatric conditions	2.62
Cardiovascular	0.03
Skin diseases	0.04
Digestive disease	0.09
Refractive errors	0.01
Endocrine disorders	0.05
Cerebrovascular disease	0.08
Malignant neoplasms	0.01
Injuries	12.98
Total	27.14

## Discussion

The WHO actively encourages the use of evidence-based health information to set priorities in health programs and policies globally [11]. Despite a recent shift of attention toward the need for acute care services, there is a paucity of empirical evidence as to how great the burden of emergency presentations actually is, and if the type of conditions may be amenable to task shifting. This study attempts to elucidate a model whereby we may quantify the burden of emergency disease and consider addressing it as a public health concern.

The burden of disease estimated by our calculations is 27 DALYs per year. At first glance, this number may seem to be low and thus not a significant burden on a population. However, because of the lack of access to care, we believe it is more accurate to compare this burden of disease to that of programs traditionally known to be high burden in LMICs. For instance, the DALYs for HIV, tuberculosis, and malaria in Fort Liberte are 4.0, 0.06, and 0.01 by comparison. Therefore, these three diseases combined contribute five times fewer DALYs to the burden of disease but receive 225 times more funding [12-14].

Users of the ER in Fort Liberte are in the most productive years of their lives; disability in this age group is particularly devastating. It is important to note the significant amount of relatively young individuals that would benefit from the addition of emergency services. Trauma holding the top position for the number of ER cases underscores the importance of a first responder infrastructure that can adequately address the urgent care needs in this area [15-17]. Basic trauma needs may be met by lay-person providers and task shifting may be a feasible, sustainable, and scalable way to address this burden in a cost-effective manner [7,8].

Many limitations occurred because of the way the hospital medical records are kept. Records are written into registers by the hand of the doctor and then filed by a clerk in the hospital archives. Diagnosis is usually clinical but there may be confirmation of the diagnosis if a test exists for the disease, as for malaria, and if the patient can afford the test. There is no consistency in whether or not the patient receives a test. The ER is alternately staffed by many Cuban or Haitian doctors. Given the subjective nature of clinical diagnoses, it is difficult to assess the correlation in diagnoses among doctors. The variation in clinical testing and diagnosis add some uncertainty to the types of diagnoses recorded. There is no classification system used for the diagnosis. Differential misclassification bias may be present due to our attempt to categorize similar disease processes; it is difficult to know which way our estimates are pulled from the true value because we cannot correlate the clinical practice of the myriad of doctors to any objective standard.

Records are filed rather haphazardly, resulting in several missing months. Per the archive clerk, the months after the earthquake saw a large increase in patient load and doctors staffing the ER; records were sometimes not kept and sometimes misplaced. The loss of records in some of the months subsequent to the earthquake may contribute some selection bias to the study design. However, the purpose of this study is to assess the baseline need for emergency care services. Patient volume at the end of 2010 trends toward the baseline patient volume of 2009. Thus, not including the aftermath immediately after the earthquake is likely a more realistic description of the need in Fort Liberte.

The results of the study may not be generalizable to all Haitian towns depending on the development of the town and its resources. Fort Liberte is similar to most mid-size Haitian towns but may differ from the two large cities in Haiti, Port-au-Prince and Cap-Haitien. For example, the amount of road traffic likely influences the frequency of trauma-related complaints and would be greater in the large cities. However, we believe that our results are generalizable to many mid-size Haitian towns.

The DALY calculation is an estimate based on many assumptions that add uncertainty to its valuation. The cross-sectional nature of the study may have complicated the DALY calculation, as there was no total duration of disease available. Instead of direct calculation, we indirectly estimated the DALYs by taking total DALYs of the disease and multiplying by the proportion of people seen for that disease in the ER. The estimation of DALYs assumes that Fort Liberte mirrors the disease burden of the larger Haitian population. Regional variations in disease frequency may exist, skewing the data.

In counting emergency presentations, we treated each visit as unique. In truth, some visits may be from the same person, particularly for chronic diseases prone to acute complications like diabetes or HIV. Also, for chronic diseases, the original GBD takes into account the long-term disability of the disease whereas an acute complication may be limited because it can be cured. For example, diabetic ketoacidosis (DKA) is an acute, curable presentation of diabetes. Using the GBD DALYs for diabetes does not reflect the shorter duration of DKA as GBD is a product of disability weight and time. The severity of DKA is considerably more than diabetes mellitus (DM) though, potentially balancing some of the overestimation from time affected by the underestimation in disability weight. Furthermore, due to the economic constraints, and the lack of readily accessible care, inhabitants of Fort Liberte and surrounding areas often defer seeking care. Therefore, the number of people visiting annually multiplied by the DALY per capita of the disease is likely an underestimation of the disease burden in the community, failing to account for those who cannot access care.

Another area of uncertainty is that we assigned all unrelated diagnoses, except for trauma, equal weight. That is, in total, we counted the charts with multiple diagnoses as a unique contributor to disability. For example, if a final diagnosis was hypertension (HTN) and DM, we counted that visit in both HTN and in DM. A widely acknowledged shortcoming of the 2004 GBD is that it does not have a method to combine the disability of separate but related diagnoses such as DM and HTN [8]. We felt that each such diagnosis adds to the disability a person suffers and should be counted separately. In the case of trauma, however, we did not do so; this is because for most mechanisms of trauma, like road traffic accident, it is common for multiple injuries to occur and counting each separately would have led to a gross overestimation.

Finally, our selection of emergency visits does demonstrate some selection bias. Obstetric emergencies could not be counted in our study because the obstetric portion of the hospital is completely separate, and the emergency visits were not separated out from the clinic visits. Obstetric emergencies are a significant contributor to the burden of disease so our estimation is likely lower than the true burden of emergency diseases in Fort Liberte. Furthermore, the money required for consultation in the ER may make it more likely that the poorest in the community will never come to the ER, even for an emergency. Death may occur outside the hospital; death records were only available for people brought to the hospital. Those cases could not be counted in our study. Thus, the true estimate of burden of disease of emergency conditions is likely underestimated by this methodology.

## Conclusions

This paper quantified the relative burden of emergency disease using a novel method that may be used to fill the data gap regarding resource allocation for emergency programs. In comparison to the commonly funded public health concerns of HIV, TB, and malaria, a sufficient emergency burden of disease affects population health in Fort Liberte, Haiti to warrant addressing it as a public health concern. Further, the types of conditions in this review show that task shifting may be a feasible way to address this emergency burden of disease.

## Appendices

Appendix 1: example of DALY estimation

Estimating annual emergency DALYs from respiratory infection (RI): 118 people presented to the ER with acute respiratory infections over 19 months.

To estimate the annual incidence:

(118/19)*12 = 74.52 people/year

DALYs/capita of respiratory infections in Haiti:

DALYs RI in Haiti/population Haiti

313,000/9,149,000 = 0.0342

Annual emergency DALYs from RI:

(DALYs RI/capita)*(annual ER visits for RI in FLH)

(.0342)*(118) = 2.55

DALY estimation for malaria in Fort Liberte: annual incidence of malaria in Haiti is 36,774.14. The population of Haiti is 9,149,000 and of Fort Liberte is 24,232.

Annual incidence of malaria in Fort Liberte is:

(36,774/9,149,000)*24,232 = 97.4

DALYs malaria/capita in Haiti

(14,000/9149,000) = 0.0015

Annual DALYs from TB in Fort Liberte:

(.0015)*(97.4) = 0.149
